# Supplementary material for: Metabolomics to Exploit the Primed Immune System of Tomato Fruit
Source: Metabolites. 2020 Mar 6;10(3):96. doi: 10.3390/metabo10030096 (PMC7143431; doi:10.3390/metabo10030096)
Supplement: Supplementary file 1 [file metabolites-10-00096-s001.zip › Figure S1 - S3.pdf]

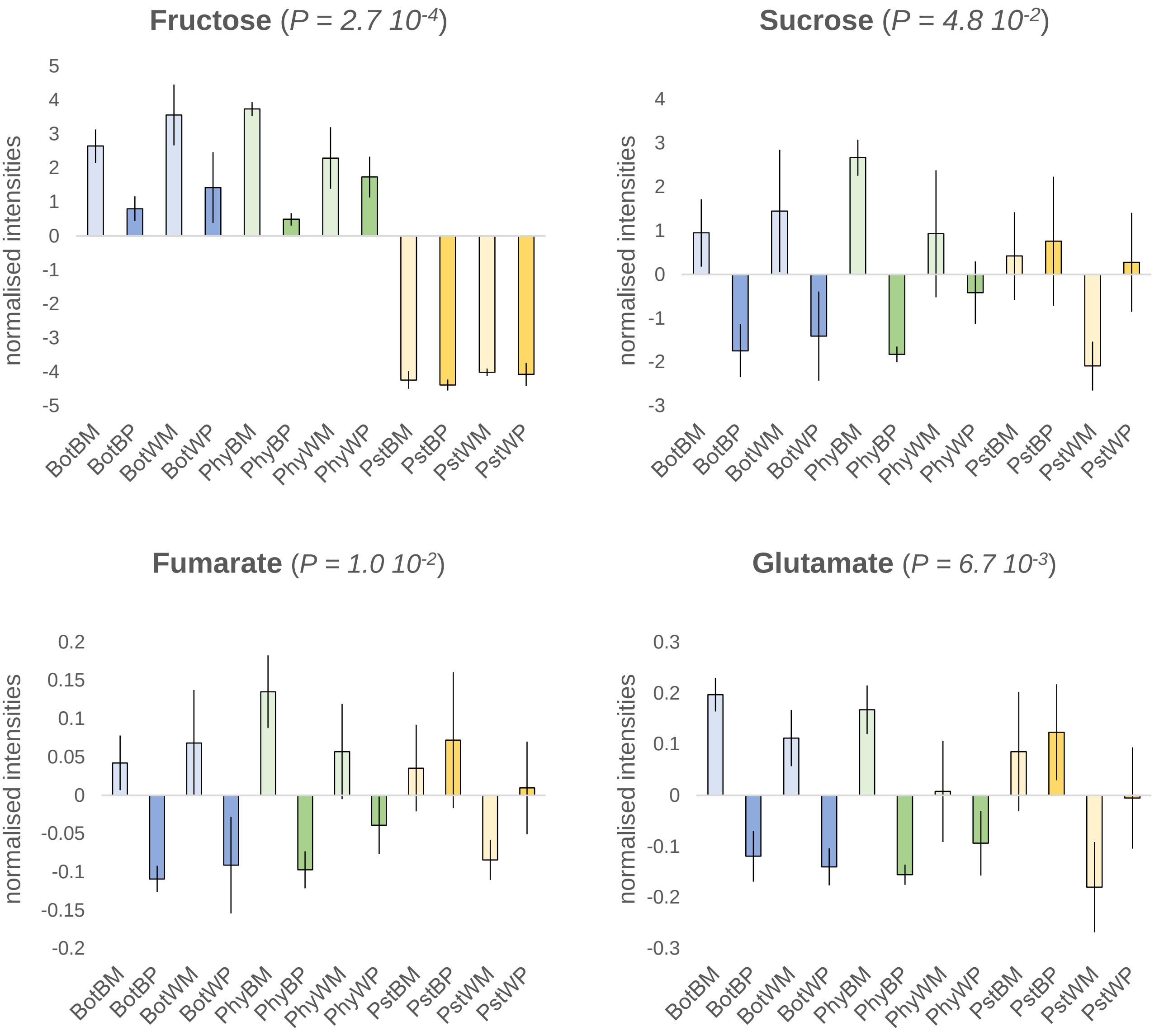

Figure S1. Inoculation responsive central metabolites. Major compounds involved in central metabolism statistically responded to the inoculation factor of a two-factor ANOVA (*P* given into brackets). Bar charts indicate means of normalised intensities of 4 independent bioreplicates (*n* = 4; ± SEM). Bot: *Botrytis cinerea*, Phy: *Phytophthora infestans*, Pst: *Pseudomonas syringae* pv. *tomato*, B: BABA-treated plants, W: water-treated plants, M: mock-inoculated fruit, P: pathogen-inoculated fruit.

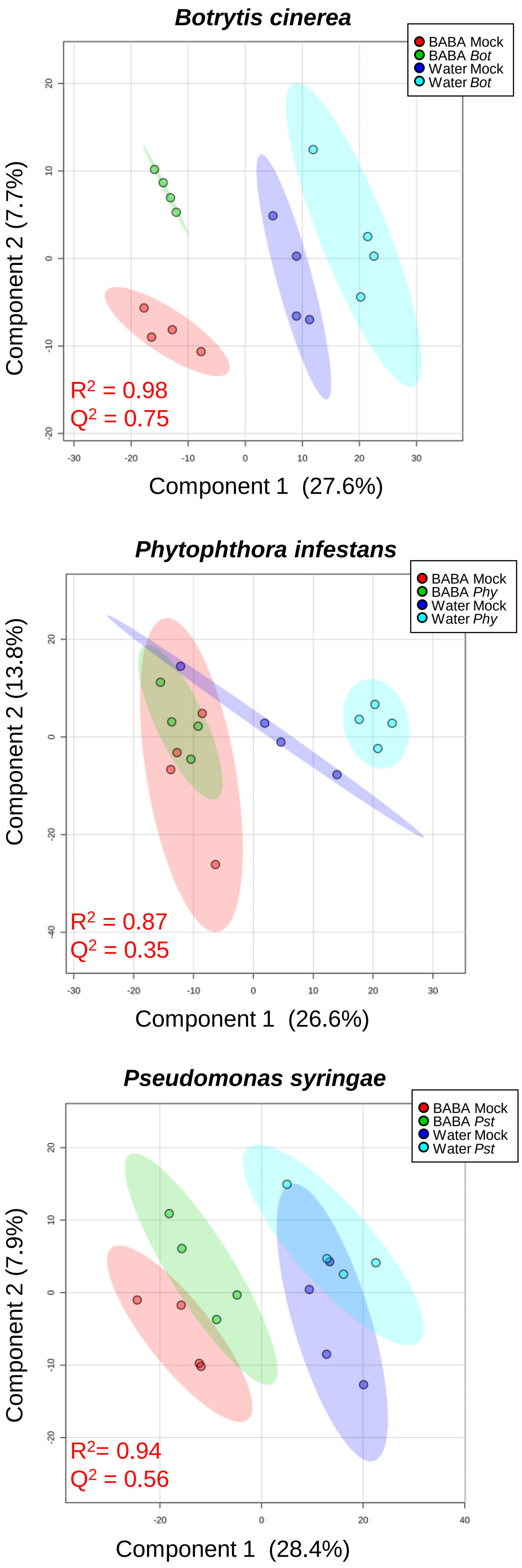

Figure S2. Partial least square discriminant analysis for each pathosystem. PLS-DA score plots (n = 4) of 6,898 features (6,887 LCMS variables + 11 major compounds) between the three different pathosystems. Validation parameters of the PLS model are given in red for each plot.

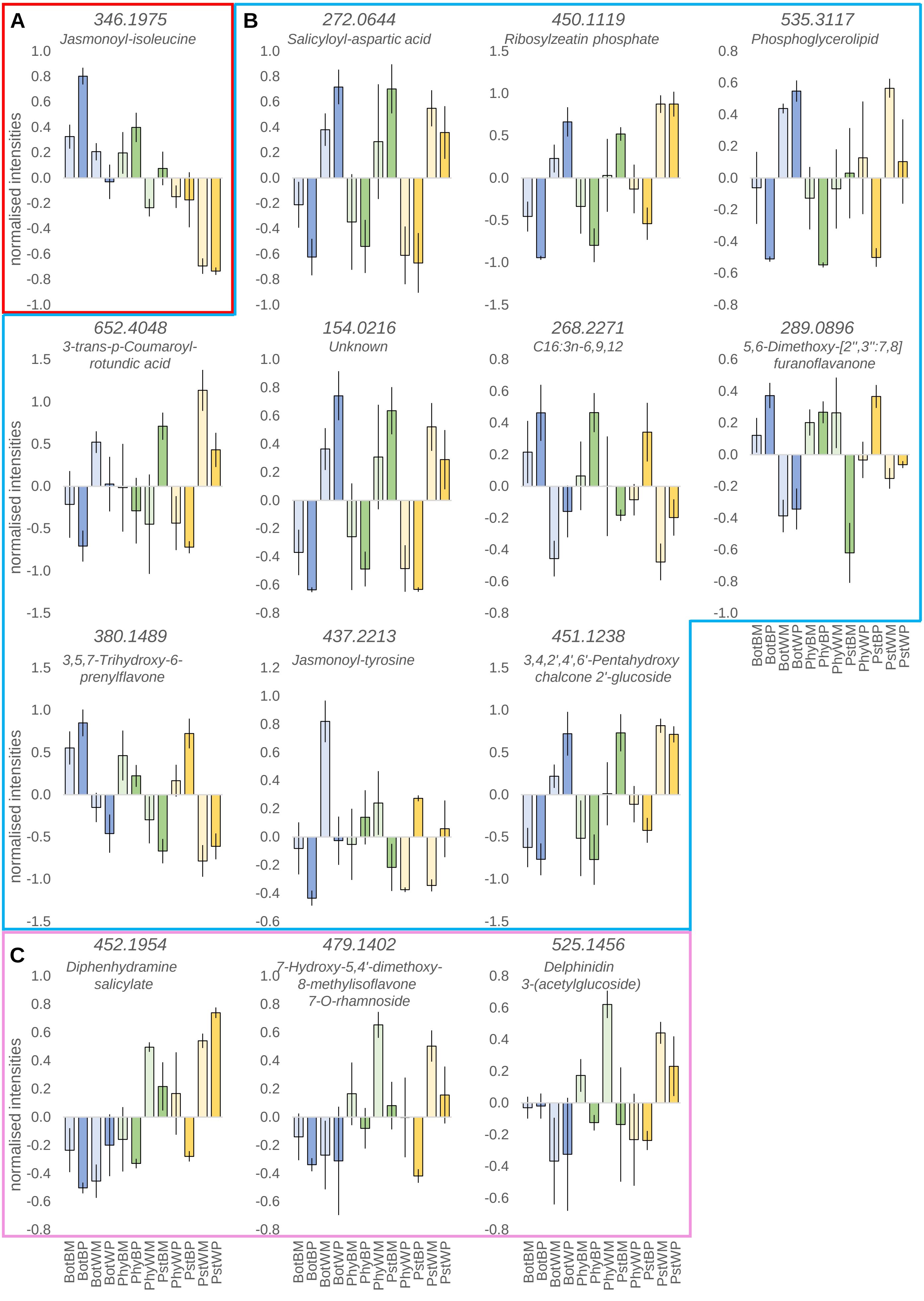

Figure S3. Metabolic markers for BABA primed responses against fruit pathogens. LCMS significant markers that overlap between *Bot* and *Phy* (**A**), *Bot* and *Pst* (**B**) and *Phy* and *Pst* (**C**) in response to BABA priming and after infection (see Table 3). Markers are labelled according to their high-resolution detected *m/z*. Bar charts indicate means of normalised intensities (n = 4; ± SEM). See Figure S1 for sample labels.
